# StepBrain: A 3-Dimensionally Printed Multicompartmental Anthropomorphic Brain Phantom to Simulate PET Activity Distributions

**DOI:** 10.2967/jnumed.123.267277

**Published:** 2024-09

**Authors:** Maria Agnese Pirozzi, Valeria Gaudieri, Anna Prinster, Mario Magliulo, Alberto Cuocolo, Arturo Brunetti, Bruno Alfano, Mario Quarantelli

**Affiliations:** 1 Department of Advanced Medical and Surgical Sciences, University of Campania “Luigi Vanvitelli,” Naples, Italy;; 2 Department of Advanced Biomedical Sciences, University of Naples “Federico II,” Naples, Italy;; 3 Institute of Biostructures and Bioimaging, National Research Council, Naples, Italy; and; 4 Human Shape Technologies Srl, Naples, Italy

**Keywords:** anthropomorphic brain phantom, PET/CT, 3D printing, ^18^F-FDG

## Abstract

An innovative multicompartmental anatomic brain phantom (StepBrain) is described to simulate the in vivo tracer uptake of gray matter, white matter, and striatum, overcoming the limitations of currently available phantoms. **Methods:** StepBrain was created by exploiting the potential of fused deposition modeling 3-dimensional printing to replicate the real anatomy of the brain compartments, as modeled through ad hoc processing of healthy-volunteer MR images. **Results:** A realistic simulation of ^18^F-FDG PET brain studies, using target activity to obtain the real concentration ratios, was obtained, and the results of postprocessing with partial-volume effect correction tools developed for human PET studies confirmed the accuracy of these methods in recovering the target activity concentrations. **Conclusion:** StepBrain compartments (gray matter, white matter, and striatum) can be simultaneously filled, achieving different concentration ratios and allowing the simulation of different (e.g., amyloid, tau, or 6-fluoro-l-dopa) tracer distributions, with a potentially valuable role for multicenter PET harmonization studies.

## Visual Abstract

**Figure:**
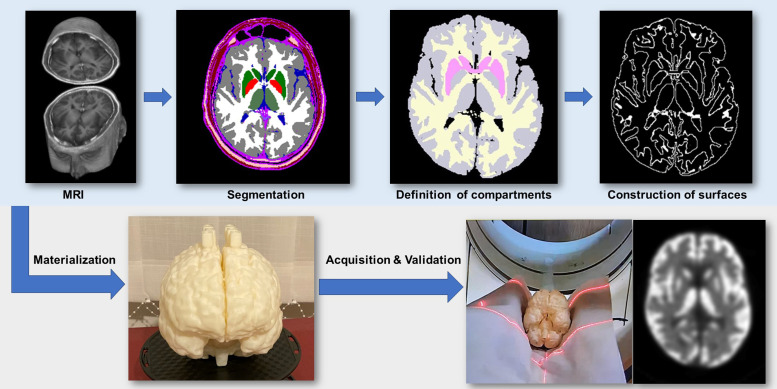

Brain imaging phantoms have been used to assess PET acquisition, reconstruction, and postprocessing inaccuracies (1*,*^2^). Compared with geometric phantoms, anatomic ones can help capture the complexity of brain structures and are useful in testing nuclear medicine systems by providing a simulation of the in vivo activity distribution of tissues (1*,*^3^–^5^).

Three-dimensionally printed phantoms can be designed with multiple compartments, also providing an accurate anatomic definition (6*,*^7^). Furthermore, a challenging task is to create separate fillable compartments, also allowing an accurate simulation of the respective radiotracer distributions (8).

StepBrain (Human Shape Technologies Srl) is an innovative anthropomorphic brain phantom for multimodal imaging studies, obtained using fused deposition modeling 3-dimensional (3D) printing technology. It consists of 3 separate compartments and can simultaneously simulate both morphologic details and tracer concentration differences in gray matter (GM), white matter (WM), and the dorsal striatum (caudate nucleus and putamen).

This work explored whether StepBrain simulation of an ^18^F-FDG PET/CT study provides results with a level of verisimilitude not achieved before.

## MATERIALS AND METHODS

### Design

StepBrain was obtained from a digital phantom (voxels, 0.9375 × 0.9375 × 1 mm) derived from multiparametric segmentation (9*,*^10^) of the MR images of a 38-y-old male volunteer (http://lab.ibb.cnr.it/Phantomag_Desc.htm) (11). Cerebrospinal fluid and extracranial tissues were preliminarily merged with the background, leaving only the brain parenchyma voxels. The deep GM nuclei (pallidum, dentate nucleus, and thalamus) were associated with the GM compartment, whereas the substantia nigra and red nucleus were associated with the WM. The caudate nucleus and the putamen were included in a separate compartment defining the dorsal striatum. Left and right caudates and putamen were transformed into a single compartment adding cylindric connections (lumen, 5 mm).

Voxel islands of GM or WM, 3-dimensionally disconnected from the main corresponding tissue compartment, were assigned to the surrounding prevalent tissue.

For each compartment, 2 bilateral cylindric pipes for filling (lumen, 6 mm) were added to the model, reaching from the top of the apical surface of each compartment.

Subsequently, nearest-neighbor interpolation was performed to obtain voxels of 0.75 × 0.75 × 0.75 mm, to model submillimeter-thick walls, still compatible with the minimum wall thickness achievable by fused deposition modeling 3D printing after optimization of the printing parameters (12).

**FIGURE 1.**
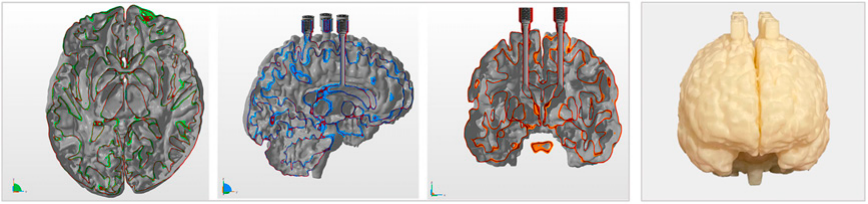
From left to right: axial, sagittal, and coronal views of 3D model, and photograph of StepBrain phantom.

Single voxelized surfaces were extracted at the compartments’ interface by selecting voxels (from the outside toward the inside) sharing vertices, edges, and faces (13), thus ensuring the extraction of a nonperforated Standard Tesselation Language model for 3D printing (12). The Standard Tesselation Language was derived using the Marching Cubes high-resolution 3D surface construction algorithm (14). The Standard Tesselation Language shells had no problems in the triangular mesh but needed further refinements to complete the phantom access via the tubes and eliminate any noise shells (Fig. 1). Finally, threaded joints were added to each tube to allow hermetic closure of the compartments after filling.

### Materialization

The phantom was materialized using the Stratasys F370 professional fused deposition modeling 3D printer. As printing material, acrylonitrile butadiene styrene was used, along with the specific material (QSR Support; Stratasys) for soluble supports (13). Advanced fused deposition modeling 3D printing settings were optimized to ensure that the printed walls had continuity, minimizing the presence of microholes and air gaps in the same walls (12). Printing the brain phantom took 7 d 3 h. After printing, support structures were removed by 3 d in an agitated bath (4.5% NaOH, at 70°).

To verify complete cleaning of internal supports, a high-resolution CT scan of the empty phantom was obtained, which was also used for measurement of compartment volumes (“Phantom Loading” section) and for definition of compartment maps for partial-volume effect (PVE) correction (“PET/CT acquisitions and processing” section).

To guarantee the impermeability of the outermost compartments, so that, once filled, the liquid and radiotracer would not cross from one compartment to another, or outward, the phantom was treated with a polyvinyl acetate solution that could penetrate the print weft by physically closing the micropores in the printed surfaces.

### Phantom Loading

The typical GM/WM contrast of ^18^F-FDG PET studies was simulated. Accordingly, the reference ratios between GM, WM, and striatum were preliminarily calculated on the basis of data reported by Greve et al. (15). To this end, for each compartment the average SUV was age-corrected to the age (38 y) of the volunteer represented in the phantom, resulting in a GM/WM ratio of 2.80 and a striatum/WM ratio of 2.63.

Phantom compartments were preliminarily filled by immersion in water containing 0.05% surfactant and continuously manually rotated for approximately 20 min to eliminate air bubbles. Because the compartment volumes, calculated by manual segmentation of the high-resolution CT images, were 710.7, 408.3, and 14.6 mL for the GM, WM, and striatum, respectively, 51, 10.7, and 1.1 MBq were then injected into the 3 compartments, respectively, and allowed to freely diffuse for 30 min, during which the phantom was placed on a dedicated rotating system to favor a homogeneous diffusion of the radiotracer within each phantom compartment.

### PET/CT Acquisitions and Processing

The PET/CT acquisition of StepBrain was performed at the Nuclear Medicine Unit of the University of Naples “Federico II” on an Ingenuity TF-128 PET/CT scanner (Philips) using the acquisition and reconstruction protocol routinely applied for clinical ^18^F-FDG studies in adults. The spatial resolution of the scanner is 4.8 mm in full width at half maximum at the center and 5.1 mm at 10 cm, resulting in 6.25 and 6.48 mm, respectively, for images reconstructed with the reconstruction filter routinely used for clinical studies (4 mm in full width at half maximum) (16).

The PET/CT images were processed using PVELab software (17) (release 2.2, https://github.com/swederik/pvelab), assuming a uniform resolution of 6.36 mm in full width at half maximum (mean value in 10-cm field of view). To this end, the automated anatomic labeling atlas (18) was applied to the high-resolution CT images previously manually segmented to provide the reference GM, WM, and striatal volumes, which in turn were coregistered to the CT scan of the PET/CT study, thus providing an automatically parcellated GM map registered with the PET images. All the different region-of-interest–based and voxel-based PVE correction methods implemented in PVELab were then applied to obtain PVE-corrected values for GM, WM, and the GM regions of interest.

For visual comparison, a PET/CT acquisition of the widely used Hoffman brain phantom (Data Spectrum Corporation) (1) was also obtained.

## RESULTS

**FIGURE 2.**
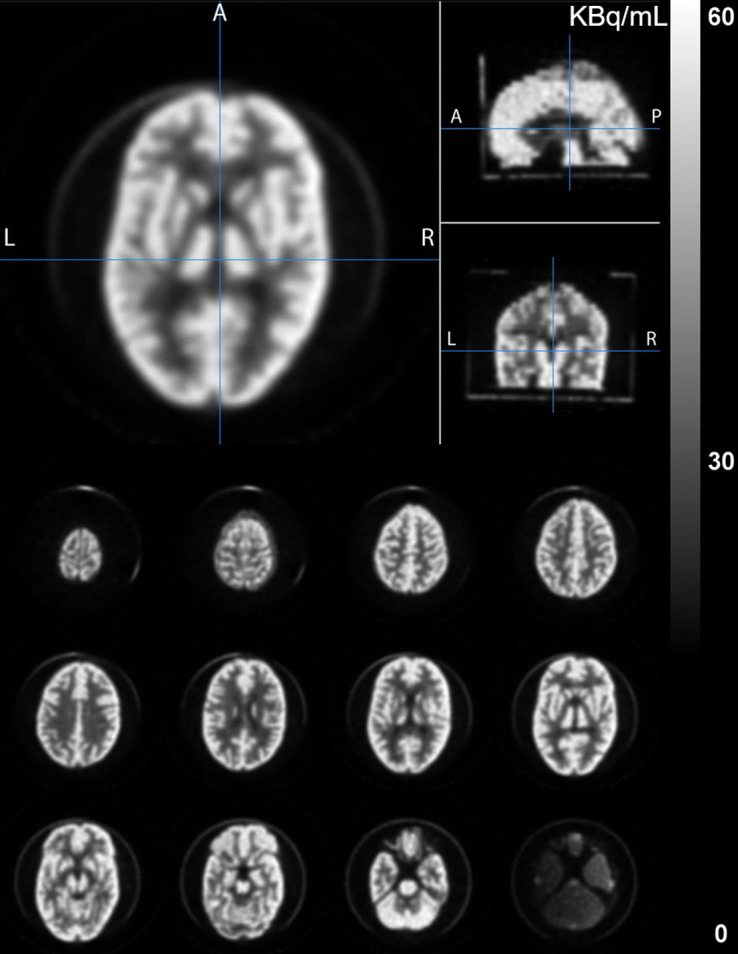
PET images of Hoffman brain phantom. A = anterior; P = posterior.

**FIGURE 3.**
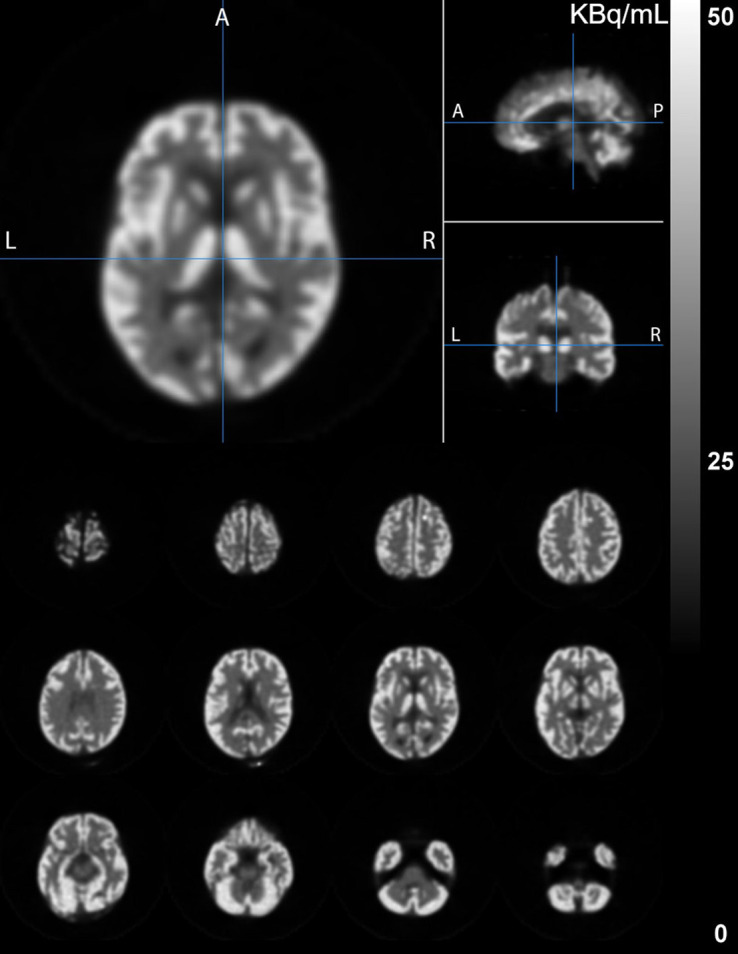
PET images of StepBrain phantom. A = anterior; P = posterior.

Figure 2 shows the PET images of the Hoffman brain phantom, and Figure 3 shows the PET images of the StepBrain phantom.

**FIGURE 4.**
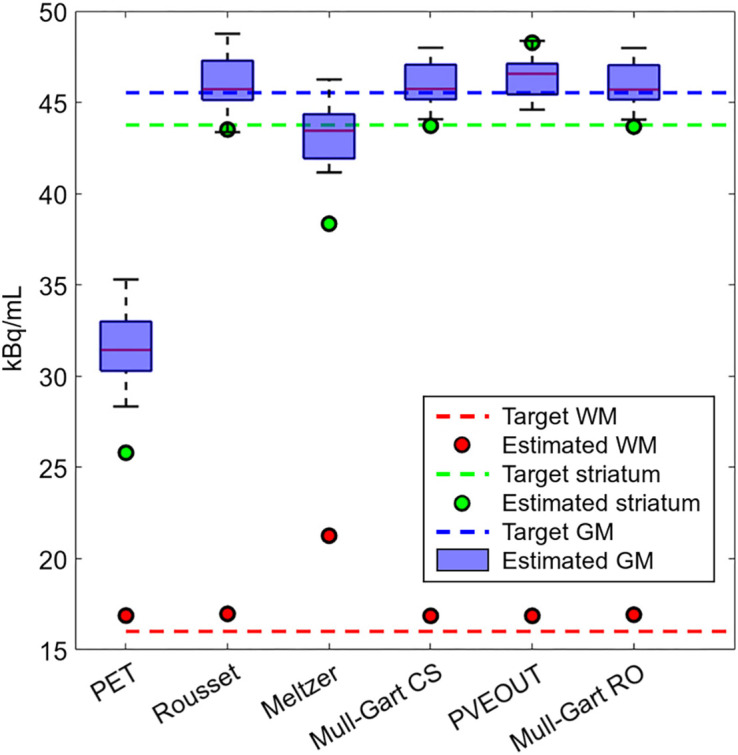
Activity concentrations derived from StepBrain PET (GM counts averaged over GM regions of interest). Concentrations are reported both uncorrected (PET) and PVE-corrected using methods proposed by Meltzer, Rousset, Alfano (PVEOUT), and Muller-Gartner with WM activity measured over Centrum Semiovale (Mull-Gart CS) or according to Rousset (Mull-Gart RO), as implemented in PVELab (17).

From the doses injected into the volumes of GM, WM, and striatum, the number of theoretic counts expected for each compartment at scanning time were calculated (target values in Fig. 4 and Table 1). PVE-corrected data using the different PVE correction methods available in PVELab (17*,*^19^) are reported in Figure 4 and Table 1.

**TABLE 1.** Uncorrected (PET) and PVE-Corrected Counts Derived from StepBrain PET (GM Counts Averaged over GM Regions of Interest)

Compartment	Target	PET	Rousset	Meltzer	Muller- Gartner CS	PVEOUT	Muller- Gartner RO
GM	45.53	31.64 ± 1.98	45.97 ± 1.45	43.30 ± 1.42	46.00 ± 1.16	46.36 ± 1.10	45.98 ± 1.16
WM	16.01	16.88	16.98	21.25	16.86	16.86	16.93
Striatum	43.76	25.80	43.51	38.35	43.72	48.27	43.67

Counts (kBq/mL) measured in GM regions of interest, WM, and striatum compartments are reported both uncorrected (PET) and PVE-corrected using methods proposed by Meltzer, Rousset, Alfano (PVEOUT), and Muller-Gartner with WM activity measured over Centrum Semiovale (Mull-Gart CS) or according to Rousset (Mull-Gart RO), as implemented in PVELab (17), along with true concentrations (Target).

## DISCUSSION

StepBrain allowed realistic simulation of the ^18^F-FDG distribution in GM, WM, and striatum, generating ^18^F-FDG–like PET studies that could be processed with tools used for human studies. The results of PVE correction confirmed the accuracy of these algorithms in recovering the tracer concentrations (apart from the well-known insufficient recovery for the Meltzer algorithm (17)), as shown by the overlap with target values and by the reduction in the SD of the GM regions of interest (which under ideal conditions should be perfectly homogeneous).

Advantages over other available brain phantoms include the presence of 3 separate compartments that can be simultaneously filled with different, arbitrarily chosen, concentrations of radiotracer, thus replicating any chosen ratio of concentrations.

The most widely available anthropomorphic brain PET phantom, the Hoffman phantom, consists of a single compartment that has a brain-shaped cavity with flat acrylic glass inserts that occupy it partly (to simulate WM) or completely (to simulate cerebrospinal fluid). Therefore, with the Hoffman phantom, a PET effect is indeed obtained by exploiting the PVE, and it works only if the PET scanner resolution along the *z*-axis is low enough not to show the layers that make up the internal slices.

Indeed, as the StepBrain phantom does not rely on PVE to obtain PET-like images, it provides realistic PET image profiles also along the *z*-axis, whereas the Hoffman phantom’s coronal and sagittal images display irregularities, especially when scanned with newer, high-resolution, digital PET scanners, because of the necessarily high slice thickness needed to accommodate acrylic glass inserts.

In addition, whereas the Hoffman phantom has a fixed GM/WM activity ratio, StepBrain can simulate any GM/WM ratio, in principle allowing simulation of amyloid or tau tracers also, with a different distribution from classic flow–metabolism PET studies.

In the absence of more appropriate solutions, the Hoffman phantom has been used to harmonize multicenter PET data of amyloid (20) and tau (21) tracers. However, it was pointed out (21) that for tracers whose distribution differs from the flow–metabolism tracers, a specific phantom is needed, and StepBrain might be the phantom to fill that role.

## CONCLUSION

By fully exploiting fused deposition modeling 3D printing potential, the StepBrain phantom replicates to a yet-unreached degree of verisimilitude the actual tracer distribution in the brain, allowing replication of a PET data acquisition under fully controlled conditions while imposing arbitrarily chosen tracer concentrations for GM, WM, and dorsal striatum. Whereas for the present proof-of-concept we simulated only an ^18^F-FDG PET study, the possibility to independently choose the different tracer concentrations in the 3 compartments allows, in principle, its use to simulate other tracers, including amyloid, tau, and dopaminergic innervation (e.g., 6-fluoro-l-dopa) tracers, as well as other imaging modalities, by filling the compartments with different suitable solutions (e.g., with different para- and ferromagnetic solutions for simulating MRI studies).

## DISCLOSURE

Financial support was provided by Regione Campania to Human Shape Technologies Srl (grant B61B19001210007). Maria Agnese Pirozzi, Anna Prinster, Mario Magliulo, Bruno Alfano, and Mario Quarantelli are cofounders of the startup Human Shape Technologies Srl, which is the applicant in the PCT-published report PCT/IB2023/059179 related to StepBrain. No other potential conflict of interest relevant to this article was reported.

## Notes

KEY POINTS **QUESTION:** Can a 3-dimensionally printed anatomic brain phantom simulate PET activity distributions? **PERTINENT FINDINGS:** Simulation of an ^18^F-FDG PET/CT study using the StepBrain phantom provided a yet-unreached degree of verisimilitude in the actual tracer distribution of GM, WM, and striatum. **IMPLICATIONS FOR PATIENT CARE:** Harmonization of multicenter PET studies performed on the StepBrain phantom could provide a useful tool to standardize diagnostic assessments based on PET images.
